# Case Report: Adverse reaction to butorphanol in a Collie homozygous for the *ABCB1-1∆* (*MDR1*) mutation

**DOI:** 10.3389/fvets.2025.1603375

**Published:** 2025-05-29

**Authors:** Tyler S. Nelson, Heather N. Allen, April Hardison, Erin Miscioscia, Rajesh Khanna, Elizabeth A. Maxwell

**Affiliations:** ^1^Department of Pharmacology and Therapeutics, Center for Advanced Pain Therapeutics and Research (CaPToR), McKnight Brain Institute, College of Medicine, University of Florida, Gainesville, FL, United States; ^2^Department of Small Animal Clinical Sciences, College of Veterinary Medicine, University of Florida, Gainesville, FL, United States; ^3^Department of Comparative, Diagnostic and Population Medicine, College of Veterinary Medicine, University of Florida, Gainesville, FL, United States

**Keywords:** MDR1, butorphanol, P-glycoprotein, opioid toxicity, ABCB1-1Δ

## Abstract

Certain dog breeds, particularly herding breeds like Collies, are predisposed to drug sensitivity due to the *ABCB1-1∆* (previously known as *MDR1*) mutation, which disrupts P-glycoprotein (P-gp) function. This mutation impairs drug efflux at the blood–brain barrier, leading to increased susceptibility to neurotoxic effects. While adverse reactions to P-gp substrate drugs such as macrocyclic lactones and chemotherapeutics are well documented, opioid sensitivity remains poorly understood. This case report documents a Collie that developed severe neurotoxicity, including profound sedation, ataxia, hypersalivation, and seizures, following a single 0.2 mg/kg dose of butorphanol. Symptoms persisted despite supportive care, requiring continuous naloxone administration for approximately 40 h before significant improvement. Neurotoxicological effects may have been exacerbated by metoclopramide and maropitant, known P-gp substrates. This case underscores the need for further research into opioid pharmacokinetics in *ABCB1-1∆* mutant dogs and highlights the importance of genetic screening in veterinary practice. To enhance patient safety, integration of automated alerts within electronic medical record systems is recommended to flag high-risk drugs for at-risk breeds, providing real-time warnings, dosing adjustments, and monitoring guidance. These measures could reduce adverse drug reactions and improve clinical outcomes in genetically susceptible dogs.

## Introduction

**Figure 1:**
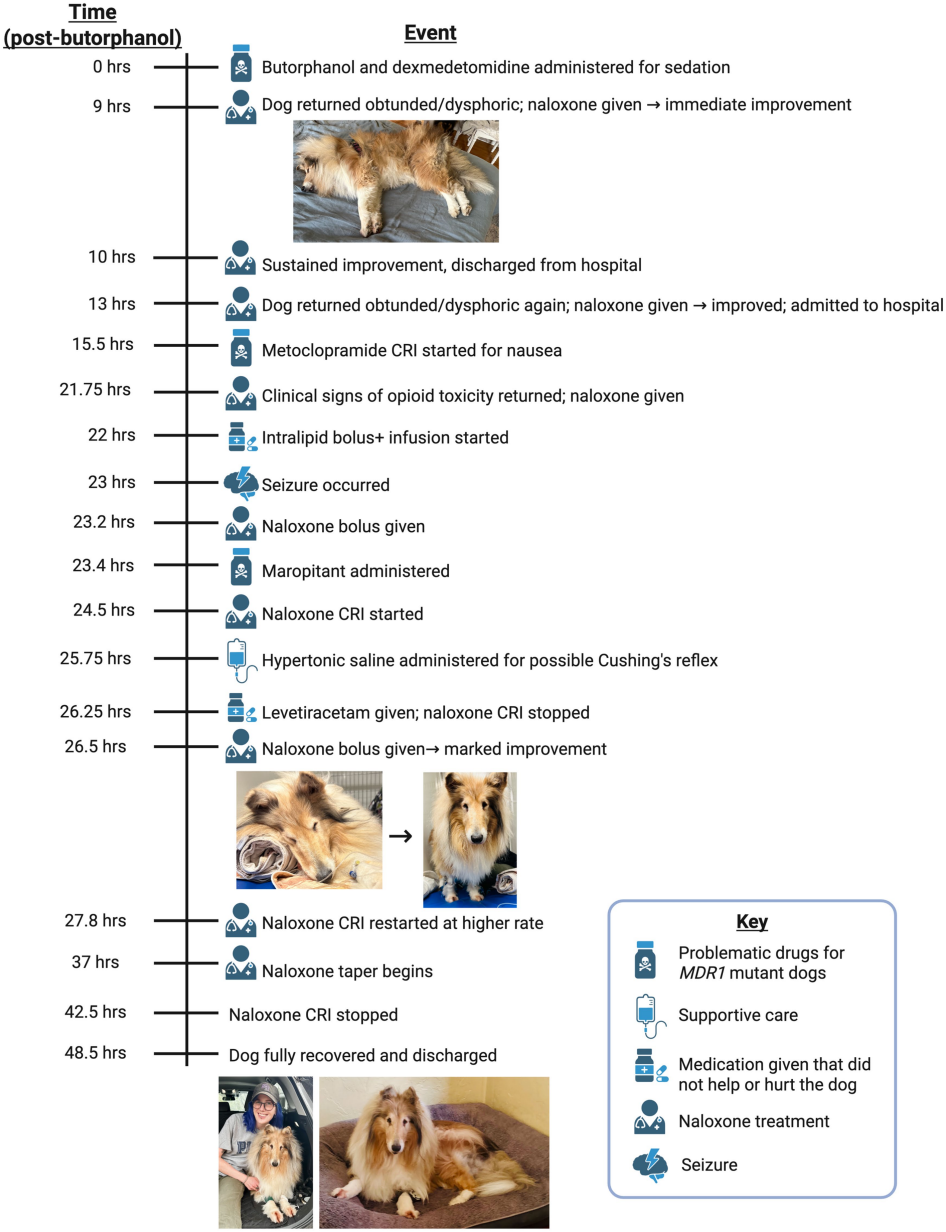
Illustrative timeline summarizing key clinical events.

Opioids are commonly used in veterinary medicine for analgesia and sedation, but herding breeds like Collies often exhibit heightened drug sensitivity due to the homozygous *ABCB1-1∆* mutation (previously known as *MDR1*) (1–3). The *ABCB1* gene encodes P-glycoprotein (P-gp), a transmembrane ATPase transporter, which plays a critical role in drug efflux at key biological barriers, including the blood–brain barrier (BBB), intestines, liver, and kidneys (4). In *ABCB1-1∆* -mutant dogs, a 4 base pair deletion in *ABCB1* produces dysfunctional P-gp transporters and impairs the body’s ability to eliminate certain drugs from the central nervous system (CNS), leading to potentially life-threatening neurotoxic effects (5–7) (Figure 1).

Known problematic drugs for *ABCB1-1∆*-mutant dogs include macrocyclic lactones (e.g., ivermectin) (8, 9), loperamide (1), apomorphine (10), acepromazine (11), and certain chemotherapeutics (12–14). However, opioid sensitivity remains poorly characterized, with anecdotal evidence suggesting that butorphanol may induce neurotoxicity in *ABCB1-1∆*-mutant dogs (7). Butorphanol, a *κ*-opioid receptor agonist and *μ*-opioid receptor antagonist, is widely used for mild to moderate analgesia and pre-anesthetic sedation in veterinary practice (15). While it is generally considered to have a favorable safety profile, its effects in *MDR1*-affected dogs remain largely unstudied.

Here a case is described of a purebred rough (bearded) Collie that developed severe opioid toxicity following routine butorphanol administration, exhibiting classic signs of opioid overdose, including profound sedation, ataxia, hypersalivation, and seizures, requiring prolonged naloxone administration. This case underscores the need for further investigation into opioid safety in *ABCB1-1∆*-mutant dogs and emphasizes the importance of genetic screening and individualized dosing adjustments in at-risk breeds.

## Case description

### History and presenting complaint

An 8-year, 7-month-old spayed female rough (bearded) Collie presented for hip radiography under sedation for evaluation of suspected osteoarthritis. The dog had a history of hindlimb stiffness, difficulty jumping, and reluctance to use stairs. Past pertinent medical history included congenital blindness (microphthalmia), gallbladder rupture and cholecystectomy, lupoid onychodystrophy, and historic adverse drug reactions. The dog received a hydrolyzed diet (Hill’s Prescription Diet z/d dry and canned) and was maintained on the following medications and supplements: s-adenosylethionine and silybin-phosphatidylcholine complex (Denamarin®, Nutramax Laboratories), lokivetmab (Cytopoint™, Zoetis Inc.), cobalamin, Dasuquin®, and fish oil.

The owner reported several historic adverse drug reactions, including prolonged recovery from anesthesia following both ovariohysterectomy and cholecystectomy. The dog historically exhibited lethargy following vaccinations, which was largely mitigated with preemptive diphenhydramine administration. Finally, 2 months prior to presentation, the dog had an adverse reaction to bedinvetmab (Librela®, Zoetis Inc.). Following a single subcutaneous (SC) bedinvetmab injection, the dog experienced two generalized (grand mal) seizures within 72–120 h, followed by the onset of significant neurological abnormalities. Postictally, the dog exhibited persistent circling to the left, pronounced motor deficits, and abnormal gait patterns consistent with central vestibular dysfunction. Over the subsequent days, caregivers reported marked lethargy, confusion, and episodes of disorientation, including becoming stuck in corners or appearing unable to navigate familiar environments. These behavioral and motor changes persisted for over a week before gradually resolving. The dog had never had a seizure prior to this incident. Bedinvetmab was discontinued following this reaction and the dog achieved a full recovery 21 days following exposure.

### Presenting physical examination

On presentation for sedated hip radiography, the dog was bright, alert, responsive, and euhydrated. Vital parameters were normal (aural temperature 99.8\u00B0F, heart rate 90 bpm, respiratory rate 24 bpm). The dog was mildly overweight (body condition score 7/9), blind and microphthalmic in both eyes. Neurologic examination was otherwise normal. The remainder of the general physical examination was also normal. The dog was ambulatory with a consistent, mild lameness of the right hindlimb, bilateral hindlimb shortened stride length, and with hindlimb stiffness. There was decreased hip abduction and decreased extension bilaterally with pain on manipulation. Mildly thickened elbows with decreased range of motion were also detected.

### Sedation and initial adverse reaction

At 0 h the dog was sedated intravenously for pelvic radiographic imaging. A low dose of dexmedetomidine (3 mcg/kg) was used due to the history of adverse drug reactions. A standard dose of the shorter-acting butorphanol (0.2 mg/kg), rather than a full *μ*-opioid, was used for added sedation and to alleviate transient pain associated with hip manipulation required for imaging. Following imaging, atipamezole (0.03 mg/kg, equal volume to dexmedetomidine) was administered intramuscularly (IM) to reverse dexmedetomidine; butorphanol was not reversed. Prior to discharge, the dog was quiet but able to weakly ambulate and posture to urinate, with normal vital parameters.

At 9 h post-butorphanol injection, the dog was re-presented with concerns of persistent sedation and abnormal respiration. On presentation, the dog was non-ambulatory, laterally recumbent, and obtunded (shown in Figure 1). An IV catheter was placed and naloxone (0.04 mg/kg IV) administered, resulting in immediate and dramatic improvement. The dog became alert, stood, and walked normally. After 1 h of observation, the dog remained bright and responsive and was discharged from the hospital.

### Recurrence of symptoms and hospital admission

At 13 h, the dog re-presented to the emergency department, again in lateral recumbency and obtunded. A second IV naloxone dose (0.03 mg/kg) was administered, again producing an immediate and dramatic recovery. Due to recurrent opioid toxicity, the dog was admitted for overnight monitoring with telemetry. At the time of admission, vital parameters were within normal limits.

At 15.5 h post-butorphanol administration the dog developed nausea, prompting initiation of a metoclopramide CRI (2 mg/kg/day IV). At 21.75 h, the dog again exhibited clinical signs consistent with opioid overdose, prompting a repeat administration of naloxone (0.03 mg/kg IV). Due to the repeated episodes of renarcotization, intravenous lipid emulsion (ILE) therapy was initiated to enhance systemic opioid clearance. A 40 mL bolus of intralipid was administered at 22 h, followed by a continuous infusion of 400 mL over 1 h. Despite these measures, the dog’s neurological status continued to deteriorate. At 23 h, a focal seizure was observed, followed by significant ataxia and altered mentation. In response to these worsening signs a subsequent naloxone dose (0.02 mg/kg IV) was given at 23.2 h and additional supportive interventions were provided as the dog’s condition progressed. Maropitant (0.5 mg/kg IV, Cerenia®, Zoetis Inc.) was administered at 23.4 h, to address signs of nausea. At 24.5 h, a continuous IV naloxone infusion was started at 0.01 mg/kg/h, and Normosol-R IV fluids were initiated. Due to bradycardia and elevated blood pressure, 90 mL of hypertonic saline was administered intravenously at 25.75 h. For ongoing seizure management, levetiracetam (57.3 mg/kg IV) was administered at 26.25 h and the continuous naloxone infusion was stopped due to concern for central neurologic disease. By this time, the dog had become non-ambulatory, stuporous, and exhibited profound neurological dysfunction despite aggressive medical management.

### Suspicion of MDR1 sensitivity and initiation of continuous naloxone infusion

Given the refractory opioid toxicity and prolonged responsiveness to naloxone, a literature review suggested that the Collie might be homozygous for the *ABCB1-1∆* (*MDR1*) mutation, resulting in deficient P-gp function and delayed butorphanol clearance (7). Another naloxone bolus (0.03 mg/kg IV) was administered at 26.5 h post-butorphanol administration and the dog exhibited a marked improvement, regaining motor coordination and responsiveness (shown in Figure 1). To mitigate drug accumulation and neurotoxic effects, the metoclopramide CRI was discontinued, and the continuous naloxone infusion was restarted and increased to 0.04 mg/kg/h IV at 27.8 h. The dog gradually showed improvement. By 37 h the naloxone CRI was tapered to 0.02 mg/kg/h, further reduced to 0.01 mg/kg/h at 40.5 h, and discontinued by 42.5 h.

### Recovery, discharge, and genetic testing

At 48.5 h post-butorphanol administration, the dog remained alert, ambulatory, and neurologically stable, allowing for safe discharge with at-home monitoring instructions. Prior to discharge, a peripheral blood sample was collected in an EDTA tube and submitted for genetic testing using the PrIMe® MDR1 genetic test, offered by Washington State University’s Program in Individualized Medicine, the leading reference center for *ABCB1-1∆* (*MDR1*) genotyping in dogs.

Testing was conducted using proprietary PCR-based methods (U.S. Patent No. US006790621B2) designed to detect the characteristic four base pair deletion in the *ABCB1* gene. The resulting report (Supplementary Figure 1) confirmed that the dog was homozygous mutant (∆/∆) for the *ABCB1-1∆* mutation, indicating complete loss of P-gp function and significantly increased susceptibility to central nervous system accumulation of P-gp substrate drugs. This definitive molecular diagnosis strongly supports the clinical conclusion of *MDR1*-associated opioid neurotoxicity in this case. The chronological timeline of events in this case is summarized in Figure 1.

## Discussion

This case highlights the severe and prolonged opioid toxicity associated with butorphanol administration in an *MDR1*-mutant Collie. The *ABCB1-1∆* mutation impairs P-gp function, leading to delayed opioid clearance, excessive CNS accumulation, and prolonged pharmacologic effects. The clinical presentation, including profound sedation, respiratory depression, ataxia, and seizures, mirrors previous reports of opioid toxicity in *ABCB1-1∆*-afflicted collies, particularly those exposed to loperamide (1, 3).

**Figure 2:**
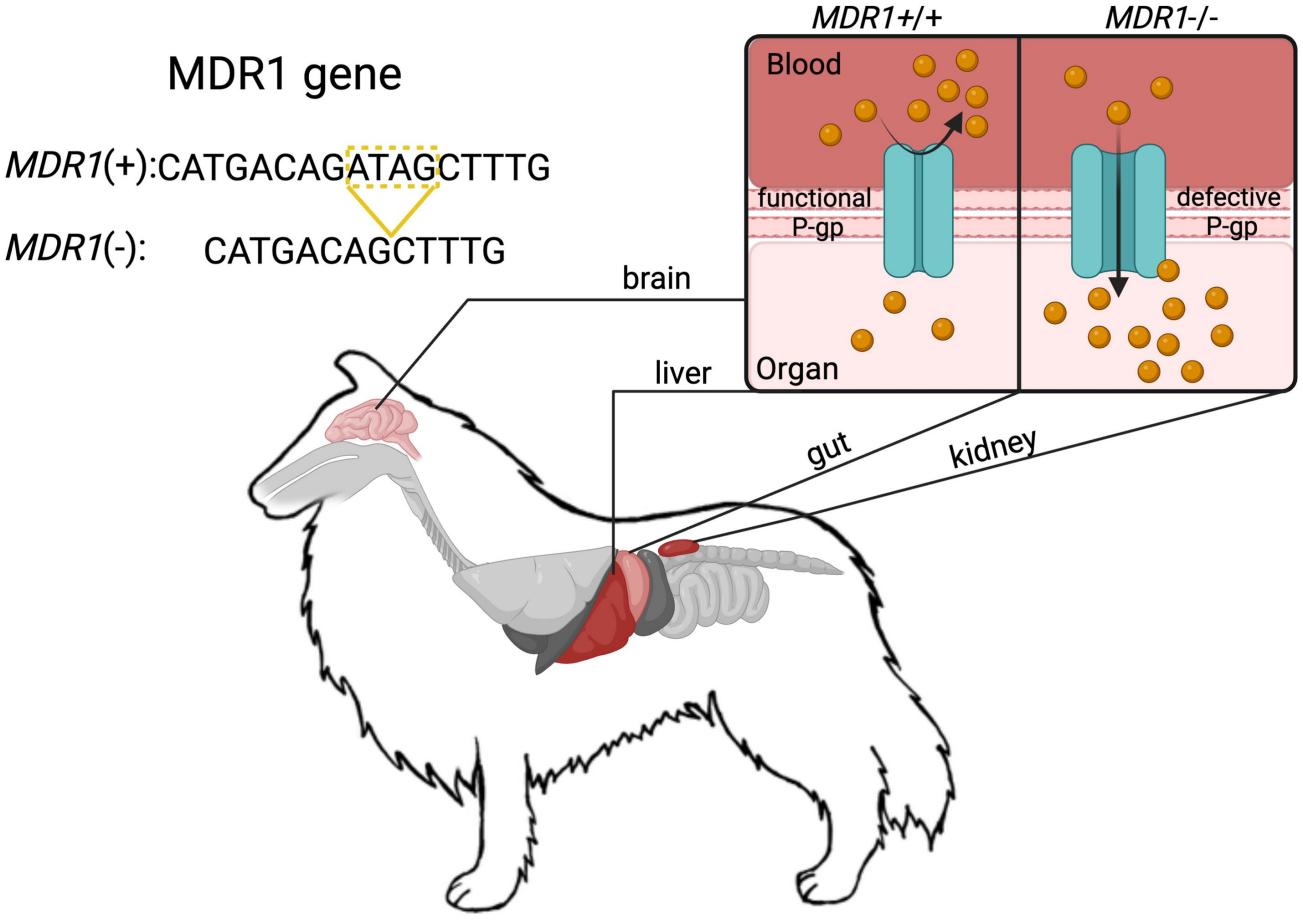
Schematic representation of the *ABCB1-1∆* (*MDR1*) mutation in Collies and the associated loss of P-glycoprotein (P-gp) efflux function at key physiological barriers. The 4-base pair deletion in the *MDR1* gene results in a truncated, non-functional P-gp transporter. This impairs active drug efflux at critical sites including the blood–brain barrier (BBB), intestinal epithelium, hepatic canalicular membranes, and renal proximal tubules. At the BBB, loss of P-gp function allows increased accumulation of P-gp substrate drugs (e.g., ivermectin) in the central nervous system, resulting in prolonged sedation, neurotoxicity, and seizures as observed in this case. In the liver and kidneys, impaired efflux reduces biliary and urinary excretion, prolonging systemic drug exposure. In the gastrointestinal tract, altered P-gp function can impact oral drug bioavailability. Together, these changes contribute to excessive drug accumulation and exaggerated pharmacologic effects in dogs homozygous for the *ABCB1-1∆* mutation. This mechanism underlies the pathophysiology described in the case and highlights the importance of P-gp in protecting against CNS and systemic toxicity.

P-gp is a critical efflux transporter that limits opioid penetration into the CNS by actively transporting drugs out of the brain and into circulation. It is highly expressed at biological barriers, including capillary endothelial cells in the BBB, hepatocytes in the liver (facilitating biliary excretion), and renal tubular cells (enhancing urinary clearance) (Figure 2). Several opioids, such as morphine, oxycodone, loperamide, fentanyl, and methadone, are *bona fide* P-gp substrates, with *in vivo* and *in vitro* studies demonstrating that P-gp inhibition or absence leads to increased brain opioid penetration and prolonged effects (16). Although butorphanol has not been definitively classified as a P-gp substrate through formal pharmacokinetic studies, clinical observations from Washington State University, an authoritative center in *MDR1*-related drug toxicity, strongly suggest that it behaves as one. In dogs with the *ABCB1-1∆* mutation, butorphanol administration has been associated with severe neurotoxic effects that closely resemble those observed with other known P-gp substrate opioids (1, 6, 7). These real-world clinical observations provide compelling, though not yet conclusive, evidence that butorphanol is likely subject to P-gp-mediated transport. However, it is important to emphasize that direct experimental confirmation of butorphanol’s interaction with P-gp is still lacking. As such, further *in vitro* and *in vivo* pharmacokinetic studies are critically needed to determine the extent of butorphanol’s transport by P-gp and to clarify its safety profile in *ABCB1-1∆*-mutant dogs.

A key observation in this case was the limited efficacy of ILE, a treatment commonly used to sequester lipophilic drugs and promote their redistribution away from the CNS. However, *in vitro* data indicate that butorphanol is poorly sequestered by ILE (~13%) compared to fentanyl (~97%), likely explaining the lack of clinical benefit in this case (17). This suggests that the primary mechanism of toxicity in MDR1-mutant dogs is impaired opioid efflux at the BBB rather than systemic redistribution, reinforcing the limitations of ILE in this context.

Additionally, the use of metoclopramide and maropitant, known P-gp substrates, may have exacerbated neurotoxicity by further impairing drug clearance. P-gp inhibition has been shown to markedly increase metoclopramide penetration into the brain (18). While not strictly contraindicated, both metoclopramide and butorphanol are classified as “Class B” drugs for dogs with the *ABCB1-1∆* mutation, meaning they should be used only under strict veterinary supervision and with caution due to the potential for enhanced central nervous system exposure (19). Similarly, maropitant has been shown *in vitro* to be a P-gp substrate in canines (20), and has been anecdotally associated with adverse neurological effects in *ABCB1-1∆*-mutant dogs (6). In this case, concurrent administration of metoclopramide and maropitant may have potentiated butorphanol’s neurotoxic effects, contributing to progressive clinical deterioration despite initial responsiveness to naloxone (6, 21). These observations underscore the importance of carefully selecting supportive therapies in genetically susceptible dogs, as unintended P-gp substrate interactions may exacerbate CNS drug accumulation and toxicity.

The absence of functional P-gp in *ABCB1-1∆-*mutant dogs has significant implications for veterinary medicine, particularly concerning the administration of opioids, macrocyclic lactones, chemotherapeutics, and other known P-gp substrates (6). *MDR1*-affected dogs experience exaggerated and prolonged drug effects, necessitating careful dose adjustments and alternative pain management strategies. In this case, extended naloxone infusion was required to counteract butorphanol toxicity, indicating that standard opioid reversal protocols may be inadequate in *MDR1*-mutant dogs. This finding highlights the need for prolonged monitoring and modified treatment approaches when opioids are administered to genetically susceptible breeds.

Routine genetic screening in veterinary practice is essential for preventing life-threatening drug reactions. The *ABCB1-1∆* mutation is prevalent in Collies, Australian Shepherds, and related breeds, making routine genotyping a valuable tool for identifying at-risk dogs (22). Increased awareness among veterinary professionals regarding drug toxicity risks in these breeds can improve safety and clinical outcomes. A practical step toward enhancing medication safety would be integrating automated alerts within veterinary electronic medical record and prescribing software. Such systems could flag high-risk drugs for predisposed breeds, providing real-time contraindications, dosage modification recommendations, and/or enhanced monitoring guidelines. Implementing technology-driven safeguards would help prevent drug-related adverse events while enabling informed, breed-specific prescribing decisions.

In conclusion, this case provides compelling evidence that butorphanol poses a significant risk to *ABCB1-1∆* (*MDR1*) mutant dogs, reinforcing the need for genetic screening, cautious opioid use, and tailored pharmacological management in at-risk breeds. The prolonged requirement for naloxone infusion underscores the potential for sustained opioid effects in these dogs, reinforcing the need for individualized dosing adjustments and extended monitoring. The limited effectiveness of ILE and the potential compounding neurotoxicity of metoclopramide further illustrate the complex pharmacokinetic challenges in *MDR1*-mutant dogs. By implementing appropriate dosing adjustments, genetic screening, automated safety alerts, and multimodal pain management, veterinarians can reduce the risk of severe opioid toxicity and improve clinical outcomes for genetically susceptible dogs. Further research is needed to clarify the pharmacokinetics of butorphanol and other pharmacological agents in *ABCB1-1∆*-mutant dogs. Controlled studies comparing plasma and CNS concentrations in affected versus non-affected dogs would provide valuable insights into the extent of drug accumulation and its clinical impact.

## Data Availability

The original contributions presented in the study are included in the article/supplementary material, further inquiries can be directed to the corresponding author.
