# Supplementary material for: Case Report: Adverse reaction to butorphanol in a Collie homozygous for the ABCB1-1∆ (MDR1) mutation
Source: Front Vet Sci. 2025 May 29;12:1603375. doi: 10.3389/fvets.2025.1603375 (PMC12159028; doi:10.3389/fvets.2025.1603375)
Supplement: Supplementary file 1 [file Data_Sheet_1.PDF]

MDR1 GENOTYPING RESULT

Ask Veterinary Expertise Question

Submitted by

Tyler Nelson

Identification Number

DN46750603

Submission

Blood Sample

Pet

Sophie

Species

Dog

Breed

Collie

Test

MDR1

Date

2/24/2025

Accession Number

103893

Test Result

MDR1 - Mutant/Mutant

Explanation of test result

Sophie is at an increased risk for drug sensitivity. Sophie has the MDR1 mutation and will pass on the mutant gene to offspring. Drugs to avoid or that must be used at reduced dosages can be found on our "Problem Drug" page (http://www.vcpl.vetmed.wsu.edu/problem-drugs). Questions regarding drugs that are not listed on the "Problem Drug" page can be submitted through our question portal (https://prime.vetmed.wsu.edu/mdr1caddie).

A current list of problem drugs for dogs with the MDR1 mutation may be found at this web address: www.vcpl.vetmed.wsu.edu

Neal Burke, Veterinary Clinical Pharmacology Laboratory Manager
US Patent Number US 6,790,621 B2.
PO Box 609, Pullman, WA 99163-0609. Phone: 509-335-3745
